# Recurrent Mumps in a Fully Vaccinated Young Adult With a Prior Breakthrough Infection: A Case Report

**DOI:** 10.7759/cureus.114304

**Published:** 2026-08-10

**Authors:** Rithika Ramadugu, Chandra Varshini Pidikiti, Alesha Cordero Peña, Angelis Almonte, Iliana Garrido Flores, Abigail Almonte, Jose L Gonzalez, Venkataramana Kandi

**Affiliations:** 1 General Practice, Kamineni Academy of Medical Sciences and Research Centre, Hyderabad, IND; 2 Medical School, Kamineni Academy of Medical Sciences and Research Centre, Hyderabad, IND; 3 General Practice, Instituto Tecnológico de Santo Domingo, Santo Domingo, DOM; 4 General Medicine, National Health Service, Santo Domingo, DOM; 5 Primary Health Care, National Health Service, Higuey, DOM; 6 General Practice, Universidad Nacional Pedro Henríquez Ureña, Santo Domingo, DOM; 7 Clinical Microbiology, Prathima Institute of Medical Sciences, Karimnagar, IND

**Keywords:** breakthrough infection, mmr vaccination, mumps, parotitis, recurrent mumps, waning immunity

## Abstract

Mumps is a vaccine-preventable viral disease that typically presents with parotid gland swelling and constitutional symptoms. Although widespread immunization has substantially reduced disease incidence, outbreaks continue to occur among vaccinated populations, raising concerns regarding waning immunity and vaccine failure. Recurrent mumps following both prior natural infection and complete vaccination is uncommon and remains infrequently reported.

We describe a 21-year-old woman who presented with a four-day history of fever, malaise, myalgia, and progressive painful swelling of the left parotid gland associated with difficulty chewing and speaking. She had received two doses of measles-mumps-rubella (MMR) vaccine according to the national immunization schedule and had a documented history of mumps infection at nine years of age, occurring after she had already completed her two-dose MMR series (vaccine-breakthrough infection).

Physical examination revealed tender unilateral parotid enlargement, cervical lymphadenopathy, and evidence of undernutrition with a body mass index of 16.7 kg/m². Laboratory evaluation demonstrated iron deficiency anemia. Further evaluation, including positive mumps RT-PCR and positive anti-mumps IgM serology, confirmed acute mumps infection, while investigations excluded alternative causes of parotid enlargement.

The patient was managed conservatively with isolation, supportive care, nutritional supplementation, and symptomatic treatment, resulting in complete clinical recovery without complications. This case highlights that recurrent mumps can occur despite prior natural infection and complete MMR vaccination. Although waning immunity and host factors such as undernutrition may have contributed to susceptibility, a causal relationship cannot be established from a single case. Continued surveillance and awareness of breakthrough infections remain important, particularly in resource-limited settings.

## Introduction

Mumps is an acute viral illness caused by the mumps virus and is characterized primarily by parotid gland swelling, fever, and constitutional symptoms [1]. The widespread use of the measles-mumps-rubella (MMR) vaccine has substantially reduced the global burden of disease; however, mumps outbreaks continue to occur in both vaccinated and unvaccinated populations [2]. Increasing reports of breakthrough infections among adolescents and young adults have raised concerns regarding waning immunity, secondary vaccine failure, and antigenic differences between vaccine and circulating viral strains [3]. Although natural infection is generally believed to confer long-lasting immunity, recurrent mumps following both previous infection and complete vaccination remains uncommon. We report a case of recurrent mumps in a 21-year-old woman with a history of childhood mumps and complete MMR vaccination, highlighting the possibility of breakthrough infection despite presumed hybrid immunity and emphasizing the need for continued clinical vigilance.

## Case presentation

A 21-year-old previously healthy woman presented during the post-monsoon season with a four-day history of progressive painful swelling over the left cheek extending to the upper cervical region. The swelling was associated with fever, headache, malaise, myalgia, and fatigue. She reported a maximum recorded temperature of 102.2°F on the first day of illness, followed by progressive enlargement of the left parotid region and worsening pain during mastication and speech, resulting in difficulty eating and speaking. The patient denied recent travel or contact with individuals with similar symptoms. She had previously received measles-mumps-rubella (MMR) vaccination according to the national immunization schedule. Notably, she had a documented history of mumps infection at nine years of age, from which she recovered without complications. She had completed both doses of her MMR vaccination series in early childhood, several years before this episode; the infection occurring at the age of nine therefore represented a vaccine-breakthrough infection rather than mumps occurring prior to, or in the absence of, vaccination. She also reported an unintentional weight loss of approximately 9 kg over the two weeks preceding presentation. Although no specific underlying medical cause was identified during evaluation, the patient was clinically undernourished (BMI 16.7 kg/m²) with laboratory evidence of iron deficiency anemia, findings that were considered potentially relevant to her overall nutritional status. There was no history of chronic illness, immunodeficiency, surgery, medication use, or allergies.

On examination, the patient was febrile (101°F) with pallor and cervical lymphadenopathy. Examination revealed diffuse enlargement of the left parotid gland with obliteration of the angle of the mandible and elevation of the ipsilateral ear lobule. The swelling was tender, particularly in the retroauricular region, and neck movements were mildly restricted because of pain. No purulent discharge was observed from Stensen’s duct. Examination of the respiratory, cardiovascular, abdominal, neurological, and genitourinary systems was unremarkable. The chronological sequence of key clinical events is summarized in Table 1.

**Table 1:** Clinical timeline of the patient

Timeline	Event
~15 months of age	First dose of MMR vaccine
4–6 years of age	Second dose of MMR vaccine — vaccination series completed
Age 9 years	Previous mumps infection (occurring after completion of the two-dose MMR series, i.e., a vaccine-breakthrough infection)
2 weeks before illness	Significant weight loss (9 kg)
Day 1	Fever and constitutional symptoms
Day 3	Development of painful parotid swelling
Day 4	Clinical evaluation and diagnostic confirmation
Day 4	Supportive treatment and isolation
Follow-up	Complete recovery

Laboratory investigations demonstrated mild anemia (hemoglobin 10.4 g/dL) with microcytic hypochromic indices and low serum iron levels (23 μg/dL), consistent with iron deficiency anemia. The total leukocyte count was 4.15 × 10⁹/L with relative lymphopenia (883.95 cells/mm³). Monocyte and eosinophil percentages were mildly elevated (10.2% and 6.1%, respectively). Thyroid function tests, fasting blood glucose, glycated hemoglobin (HbA1c), lipid profile, serum calcium, urinalysis, and most renal and liver function parameters were within normal limits (Table 2).

**Table 2:** Summary of key investigations Abbreviations: RT-PCR, reverse transcription polymerase chain reaction; IgM, immunoglobulin M; IgG, immunoglobulin G; EBV, Epstein-Barr virus; CMV, cytomegalovirus; HIV, human immunodeficiency virus; HbA1c, glycated hemoglobin.

Investigation	Result	Interpretation	Reference Range
Hemoglobin	10.4 g/dL	Mild anemia	12.0–15.0 g/dL
Peripheral smear	Microcytic hypochromic	Iron deficiency pattern	Normocytic, normochromic RBCs
Serum iron	23 μg/dL	Reduced	50–170 μg/dL
Total leukocyte count	4.15 × 10⁹/L	Mild leukopenia	4.0–11.0 × 10⁹/L
Absolute lymphocyte count	883 cells/mm³	Relative lymphopenia	1,000–4,800 cells/mm³
Monocyte percentage	10.2%	Mildly elevated	2–8%
Eosinophil percentage	6.1%	Mildly elevated	1–6%
Mumps RT-PCR	Positive	Confirmatory	Negative
Anti-mumps IgM	Positive	Acute infection	Negative
Anti-mumps IgG	Positive	Previous immunity	Negative
Parotid ultrasonography	Acute parotitis	Supportive	Normal salivary gland architecture
EBV serology	Negative	Excluded	Negative
CMV serology	Negative	Excluded	Negative
HIV test	Negative	Excluded	Negative
HbA1c	Normal	Diabetes excluded	<5.7%

A buccal mucosal swab and nasopharyngeal swab were obtained for virological evaluation. Based on the characteristic clinical presentation, laboratory findings, and confirmatory diagnostic testing, a diagnosis of recurrent mumps infection was established despite prior natural infection and complete MMR vaccination. The patient was advised to isolate for 7-10 days and was managed with supportive treatment, including hydration, antipyretics, non-steroidal anti-inflammatory drugs, glandular massage, and nutritional supplementation. Oral iron therapy was initiated for iron deficiency anemia. The patient showed progressive clinical improvement with complete resolution of symptoms and no complications during follow-up.

## Discussion

Mumps is an acute viral illness caused by the mumps virus, a member of the family *Paramyxoviridae* [1]. Although the widespread introduction of the MMR vaccine has markedly reduced disease incidence, mumps outbreaks continue to occur globally, including among highly vaccinated populations [1,2]. Recent outbreaks in North America, Europe, and Asia have highlighted the persistence of mumps despite high vaccination coverage and have raised concerns regarding waning immunity and secondary vaccine failure [3-6].

The present case is noteworthy because the patient developed recurrent mumps despite both prior natural infection during childhood and complete MMR vaccination. Natural infection has traditionally been considered to confer long-lasting immunity, while two-dose MMR vaccination provides approximately 88% protection against mumps [4]. Nevertheless, breakthrough infections have been increasingly reported among adolescents and young adults, particularly many years after completion of the vaccination schedule [6,7]. Several mechanisms have been proposed to explain mumps infection in previously vaccinated individuals. Waning immunity is considered one of the principal mechanisms proposed to explain breakthrough infections in vaccinated populations [3,8]. In addition, antigenic differences between vaccine strains and circulating wild-type strains may reduce vaccine effectiveness in certain settings. Several studies reported mumps vaccine effectiveness and evidence of declining protection with increasing time since vaccination, supporting waning immunity as a major factor underlying recent outbreaks [3,9,10].

Modeling of vaccine-derived protection suggests a mean duration of only about 27 years, considerably shorter than the durable immunity generally attributed to natural infection, which may help explain why breakthrough disease clusters among adolescents and young adults occur several years after completion of the primary series [9]. In outbreak settings, an additional (third) MMR dose has been shown to transiently reduce mumps risk by 60-78%, and is recommended by the US Advisory Committee on Immunization Practices for persons at increased risk during defined outbreaks, although this protective boost appears to wane again within one to three years and has not been adopted as a routine, population-wide strategy [11,12].

Reports of recurrent mumps following both vaccination and previous natural infection are uncommon but have been described in the literature. The occurrence of mumps in previously vaccinated individuals has become increasingly recognized, and accumulating evidence suggests that waning immunity is the primary driver of breakthrough infections and outbreaks in highly vaccinated populations [2,3,5,6]. Our case adds to the limited literature describing recurrent mumps following prior natural infection and complete vaccination and illustrates that reinfection may occur even in individuals presumed to possess immunity acquired through both natural infection and vaccination. Malnutrition remains one of the leading causes of secondary immunodeficiency worldwide and is associated with impaired humoral and cell-mediated immune responses. Although a causal relationship cannot be established from a single case, the patient's low body mass index and nutritional deficiencies may have contributed to reduced immune protection and increased susceptibility to reinfection [13].

This case highlights the importance of considering mumps in the differential diagnosis of parotid swelling even among fully vaccinated adults and those with a prior history of infection. Although recurrent infection appears uncommon, clinicians should remain aware that breakthrough infections can occur and should pursue appropriate diagnostic evaluation when clinically indicated. Continued surveillance and further research are needed to better understand the duration of protective immunity following both natural infection and vaccination and to identify factors associated with recurrent disease.

## Conclusions

This case demonstrates that recurrent mumps may occur despite prior natural infection and complete MMR vaccination, suggesting that neither natural infection nor vaccination invariably provides lifelong protection. Waning immunity, together with host factors such as undernutrition and iron deficiency, may contribute to susceptibility in certain individuals. Clinicians should therefore maintain a high index of suspicion for mumps in patients presenting with parotitis, irrespective of vaccination status or previous infection history. Continued surveillance, prompt diagnostic evaluation, and further research into the duration and determinants of protective immunity are essential to better understand and prevent breakthrough and recurrent mumps infections.
